# Aortopulmonary Septal Defect: A rare congenital cardiac anomaly

**DOI:** 10.18295/squmj.10.2023.062

**Published:** 2024-05-27

**Authors:** Suprava Naik, V.R. Sreecharan, Sudipta Mohakud, Taraprasad Tripathy

**Affiliations:** Department of Radiodiagnosis, All India Institute of Medical Sciences, Bhubaneswar, Odisha, India

We report a case of a 12-year-old girl who presented with palpitation. The patient was able to do her regular daily activities, including attending school. However, she would become dyspnoeic on exertion.

The patient was referred to a tertiary care hospital in Odisha, India, in 2022 for a computed tomography (CT) angiography. The CT angiography was done in a Siemens 256 CT scanner (Siemens, Munich, Germany) that showed cardiomegaly with dilated left atrium and left ventricle. A defect with a size of 28 mm was noted between the ascending aorta and the pulmonary artery connecting the two vessels, approximately 16 mm cranial to the aortic root [Figure 1]. The pulmonary artery and its branches were also dilated. The main pulmonary artery measured 29 mm—its right branch measured 22 mm and left branch was 19 mm. The ascending aorta was dilated near the defect. The aorta was arising from the left ventricle and the pulmonary artery from right ventricle. The pulmonary veins were showing normal drainage into the left atrium. The branches of pulmonary artery were appearing prominent near the hilum. The lung window was showing mosaic attenuation in both lung fields. Diagnosis of aortopulmonary septal defect (APSD) with pulmonary hypertension was made.

Informed consent was obtained from the patient and from her father to use her medical data for publication.

## Comment

APSD also known as aortopulmonary window is one of the rare congenital cardiac abnormalities seen in 0.5% of all congenital heart defects.^1^ It develops due to embryological incomplete separation of the common arterial trunk. This results in an abnormal connection between ascending aorta and main pulmonary artery.

APSD are classified into 3 types based on their location. Type I is the most common where the defect lies between the posteromedial wall of the ascending aorta and the main pulmonary artery just cephalad to the sinus of Valsalva. Type II defects occur in the distal part of the aortopulmonary septum adjacent to the right pulmonary artery. Type III defects are total defects involving the entire length of the aortopulmonary septum.^2^^,^^3^ The aortic valve and right ventricular outflow tract remain normal. The truncus arteriosus may be confused with APSD; however, truncus arteriosus has a truncal valve instead of two semilunar valves in APSD.

APSD can occur in isolation or in association (up to 50% of cases) with other congenital heart defects, such as interrupted aortic arch, transposition of great vessels, coarctation of aorta, tetralogy of Fallot, ventricular septal defects, atrial septal defect and tricuspid atresia.^4^ Variation of pulmonary arteries and coronary arteries have also been described in association with this defect. There was no other associated abnormality in the current case.

A chest X-ray showed cardiomegaly and increased pulmonary vascular markings as a result of left-to-right shunt. A 2D echocardiography may demonstrate the defect, aortic root and chamber enlargements. A CT angiography can accurately delineate the location of defect, its size, distance from aortic root, size of cardiac chambers, other associated anomalies, status of branches of pulmonary arteries and the lung fields.

Treatment of an aortopulmonary window depends on its size where small defects can be closed by suture closure while larger defects may require a surgical patch closure. Catheterisation can be considered when a defect is small enough to allow for device closure without causing stenosis of the great arteries or interference with the semilunar valves. Medical management includes anti-congestive medications such as diuretics and digoxin that can provide symptomatic relief without alteration of the disease course.

## Figures and Tables

**Figure 1:**
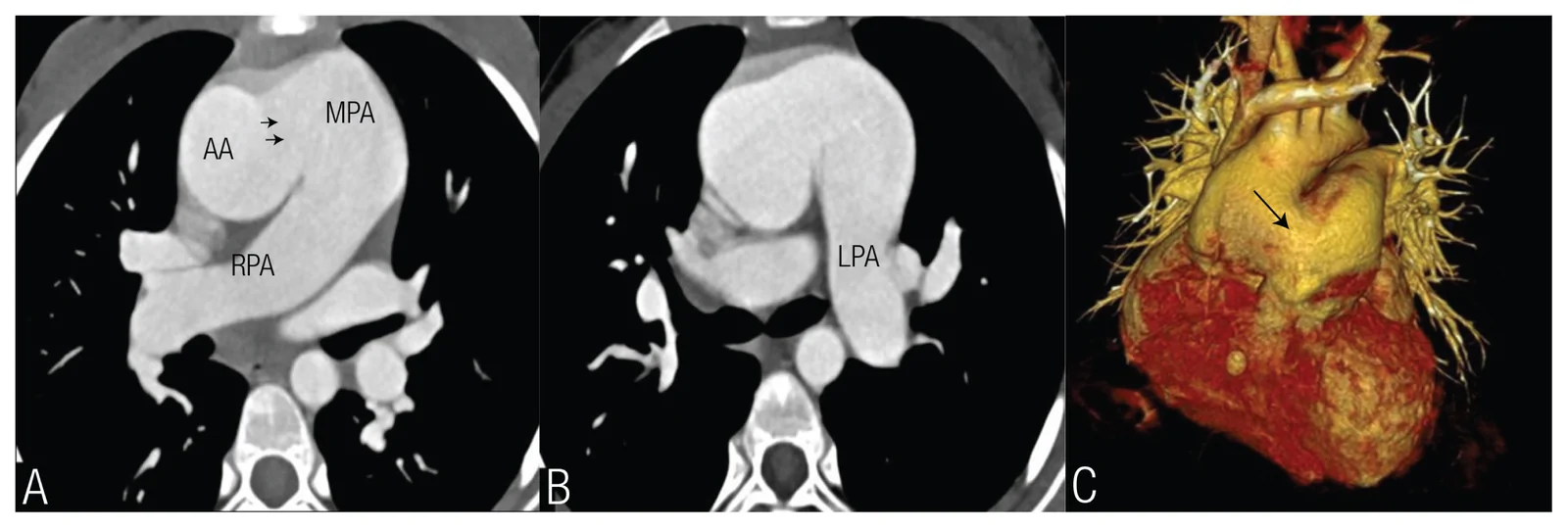
Axial sections of computed tomography angiography at the level of ascending aorta **(A and B)** showing a large defect (arrowheads) between the ascending aorta and the proximal main pulmonary artery. There is dilated main pulmonary artery, its right and left branch. Volume rendered image **(C)** showing communication between the ascending aorta and proximal main pulmonary artery (arrow) about 16 mm from aortic root. Left subclavian, left common carotid and innominate artery are arising from the arch of aorta. *MPA = main pulmonary artery; AA = ascending aorta; RPA = right pulmonary artery; LPA = left pulmonary artery*.
